# Identification of genes associated with altered gene expression and m6A profiles during hypoxia using tensor decomposition based unsupervised feature extraction

**DOI:** 10.1038/s41598-021-87779-7

**Published:** 2021-04-26

**Authors:** Sanjiban Sekhar Roy, Y.-H. Taguchi

**Affiliations:** 1 School of Computer Science and Engineering, Vellore Institute of Technology, Vellore, India; 2 Department of Physics, Chuo University, Tokyo, 112-8551 Japan

**Keywords:** Computational biology and bioinformatics, Microarrays

## Abstract

Although hypoxia is a critical factor that can drive the progression of various diseases, the mechanism underlying hypoxia itself remains unclear. Recently, m6A has been proposed as an important factor driving hypoxia. Despite successful analyses, potential genes were not selected with statistical significance but were selected based solely on fold changes. Because the number of genes is large while the number of samples is small, it was impossible to select genes using conventional feature selection methods with statistical significance. In this study, we applied the recently proposed principal component analysis (PCA), tensor decomposition (TD), and kernel tensor decomposition (KTD)-based unsupervised feature extraction (FE) to a hypoxia data set. We found that PCA, TD, and KTD-based unsupervised FE could successfully identify a limited number of genes associated with altered gene expression and m6A profiles, as well as the enrichment of hypoxia-related biological terms, with improved statistical significance.

## Introduction

Hypoxia^1^, also known as tissue hypoxia, is a serious symptom with various causes. For example, hypoxia could result in death, such as in the case of COVID-19, a serious pandemic^2^. Hypoxia also plays a critical role in cancer^3^. Both brain hypoxia^4^ and lung cell hypoxia^5^ can be fatal. Despite the significance of hypoxia, the critical factors of hypoxia are not yet fully understood^6^. Recently, m6A was reported to be a newly discovered regulator of hypoxia^7^. Wang et al.^8^ found that many genes are simultaneously associated with altered m6A and gene expression profiles in hypoxia. Although the investigations were successful, there was one methodological issue with their study; they selected genes associated with altered m6A and gene expression in hypoxia without determining statistical significance. They selected genes based on fold change (FC). Usually, only using FC to select altered expression or any other measurements might be erroneous because a sufficiently large FC might be observed simply by chance when a large number of candidates are considered. In their analysis, all human genes (as many as a few tens of thousands) and whole genome m6A were considered. In this case, if the FC was not validated statistically, a sufficiently large FC might have been observed simply by chance. The genes associated with altered m6A and gene expression based on statistical significance could not be identified because of the small number of samples; there were only four time points (including the control) measured without any replicates. If we consider the large number of genes as well as m6A peaks in the genome, it is unlikely that four samples are enough to achieve statistical significance; small samples result in larger *P*-values, whereas a large number of genes and m6A peaks result in relatively larger *P*-values. In this study, we applied principal component analysis (PCA) and tensor decomposition (TD)-based unsupervised feature extraction (FE) to select genes associated with altered m6A as well as gene expression in hypoxia to determine statistical significance. Enrichment analyses of selected genes are reasonable and consistent with previous findings^8^ and can now be supported with statistical significance. Thus, not only were the critical roles of m6A in hypoxia validated but also the usefulness of PCA-and TD-based unsupervised FE in the case where there are very few samples with a large number of variables.

There are a limited number of genomic studies using TD^9,10^. Fang proposed tightly integrated genomic and epigenomic data mining using TD^11^ (445 samples for TCGA-OV and 480 samples for TCGA-HNSC), Hore et al applied TD to multi-tissue gene expression experiments^12^ (845 related individuals), Ramdhani et al applied TD to stimulated monocyte and macrophage gene expression profiles^13^ (432 samples), Wang et al. applied TD to multi-tissue multi-individual gene expression^14^ (544 individuals), Li et al. applied TD to clinical gene-sample-time microarray expression^15^ (53 genes and 27 samples), Hu et al. applied TD to gene expression of tumor samples^16^ (more than 11,000 tumor samples), Diaz et al. applied TD to genomic data^17^ (503 patients), and Bradley et al. applied TD to DNA copy-number alterations^18^ (a few hundred samples). All methods other than that used by Li et al. included as few as 53 genes and required as many as several hundred samples, whereas our methods generally require only a few samples (in this study as few as eight samples). To our knowledge, ours is the only method applicable to a data set that includes a few samples with as many as $10^4$ genes.

## Results

**Figure 1:**
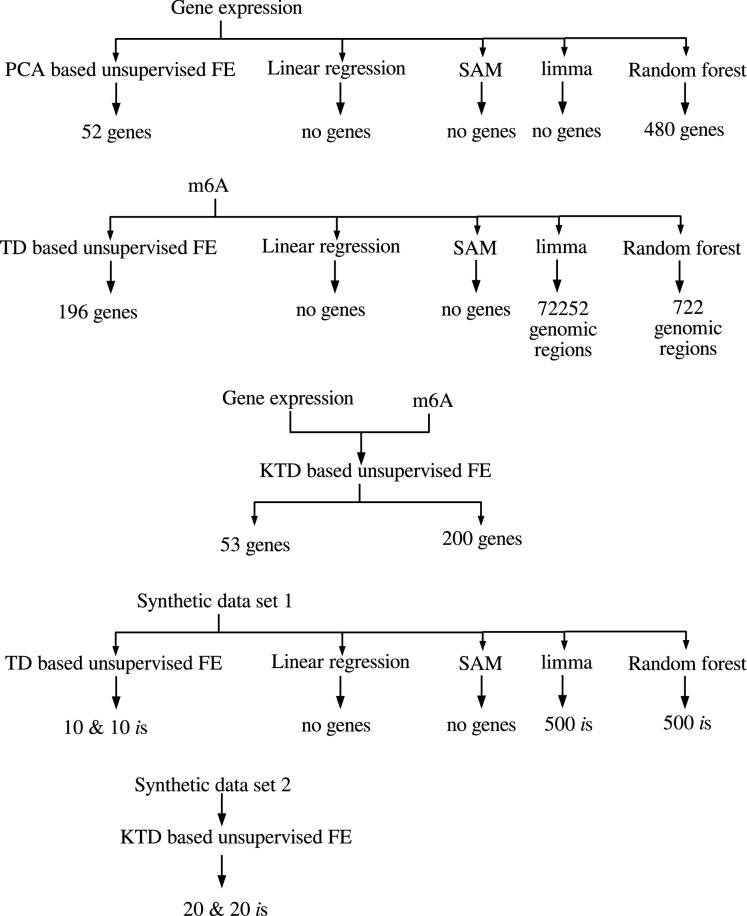
Flow chart of analyses performed in this study.

Figure 1 shows the flow chart of analyses performed in this study.

### PCA based unsupervised FE applied to gene expression profiles

**Figure 2:**
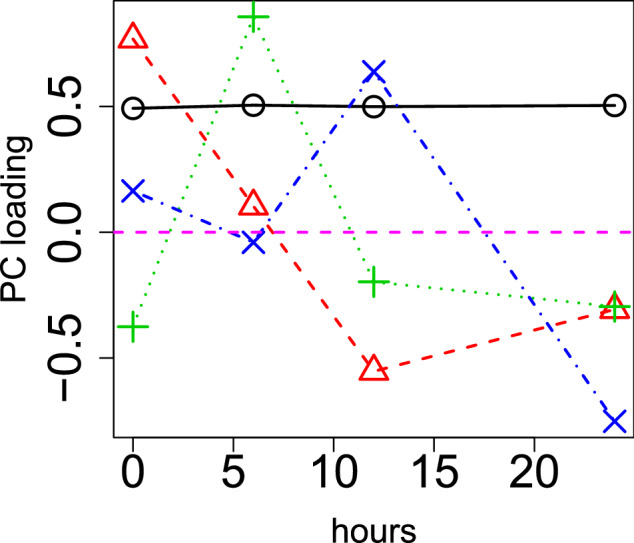
PC loading, $v_{\ell t}$, computed by PCA applied to time points by applying PCA to gene expression profiles. Open black circles: 1st, (0.61) open red triangles: 2nd (− 0.76), green crosses: 3rd (− 0.24), blue crosses: 4th PC loading (− 0.60). The numbers in parentheses are the Pearson’s correlation coefficients. Hours (horizontal axis) represent the duration after the treatments. The horizontal magenta broken line indicates baseline (zero).

Because gene expression profiles are measured solely over four time points, it is formatted as a matrix ${x_{it}} \in {{\mathbb {R}}^{N \times 4}}$, and PCA-based unsupervised FE was applied to $x_{it}$. Figure 2 shows the PC loading attributed to time points. Because the second PC loading is mostly correlated with time, we decided to employ the second PC score, $u_{2i}$, in order to attribute *P*-values to genes *i*. Using Eq. (7) with $\ell =2$, $P_i$s are attributed to gene *i*. Then 52 *i*s (genes) with associated corrected *P*-values less than 0.01, were selected. Table 1 shows the enrichment terms in “KEGG 2019 Human” categories in Enrichr for 52 selected gene symbols. Not all terms are related to hypoxia, whereas a term such as “Oxidative phosphorylation” is known to be related to hypoxia^19^. “Cardiac muscle contraction” is also known to be related to hypoxia^20^. Retrograde endocannabinoid signaling is known to be related to hypoxia^21^. Although three representative neurodegenerative diseases, “Parkinson’s disease,” “Alzheimer’s disease,” and “Huntington’s disease”, are listed, hypoxia is known to be related to neurodegenerative diseases^22^. Glycolysis is also related to hypoxia^23^. Most importantly, HIF-1, a hypoxia-inducible factor, is listed. There are additional identified enrichments that can support the success of PCA-based unsupervised FE. Although they are not always top-ranked, the 52 identified genes are also known to be up/downregulated in independent hypoxia experiments (Table 2). In the “GO Biological Process 2018” category in Enrichr, various glucose/glucogenesis related terms are enriched (Table 3). These results suggested that the analyses performed by PCA-based unsupervised FE were successful.

**Table 1:** “KEGG 2019 Human” category of Enrichr for 52 genes selected by applying PCA-based unsupervised FE to gene expression profiles. Eleven terms with adjusted *P*-values less than 0.05 are listed.

Term	Overlap	*P*-value	Adjusted *P*-value
Ribosome	19/153	$1.20 \times 10^{-27}$	$3.68 \times 10^{-25}$
Thermogenesis	13/231	$1.98 \times 10^{-14}$	$3.05 \times 10^{-12}$
Oxidative phosphorylation	11/133	$3.54 \times 10^{-14}$	$3.63 \times 10^{-12}$
Parkinson disease	11/142	$7.35 \times 10^{-14}$	$5.66 \times 10^{-12}$
Glycolysis/gluconeogenesis	8/68	$7.80 \times 10^{-12}$	$4.80 \times 10^{-10}$
HIF-1 signaling pathway	6/100	$2.27 \times 10^{-7}$	$1.16 \times 10^{-5}$
Alzheimer disease	7/171	$2.86 \times 10^{-7}$	$1.26 \times 10^{-5}$
Huntington disease	6/193	$1.05 \times 10^{-5}$	$4.05 \times 10^{-4}$
Retrograde endocannabinoid signaling	5/148	$4.07 \times 10^{-5}$	$1.39 \times 10^{-3}$
Cardiac muscle contraction	4/78	$5.03 \times 10^{-5}$	$1.55 \times 10^{-3}$
Non-alcoholic fatty liver disease (NAFLD)	4/149	$6.07 \times 10^{-4}$	$1.70 \times 10^{-2}$

**Table 2:** “Disease Perturbations from GEO down/up” category of Enrichr for 52 genes selected by applying PCA-based unsupervised FE to gene expression profiles. Six hypoxia experiments with adjusted *P*-values less than 0.05 are listed.

Term	Overlap	*P*-value	Adjusted *P*-value
**Disease perturbations from GEO down**			
Hypoxia C0242184 human GSE4630 sample 250	13/349	$3.83 \times 10^{-12}$	$2.19 \times 10^{-11}$
Hypoxia C0242184 mouse GSE3195 sample 70	10/328	$1.05 \times 10^{-8}$	$3.77 \times 10^{-8}$
Hypoxia C0242184 human GSE4483 sample 440	9/275	$3.38 \times 10^{-8}$	$1.11 \times 10^{-7}$
**Disease perturbations from GEO up**			
Hypoxia C0242184 human GSE4483 sample 440	27/325	$5.48 \times 10^{-35}$	$9.19 \times 10^{-33}$
Hypoxia C0242184 human GSE4630 sample 250	10/251	$8.07 \times 10^{-10}$	$2.38 \times 10^{-9}$
Hypoxia C0242184 mouse GSE3195 sample 70	10/272	$1.76 \times 10^{-9}$	$5.01 \times 10^{-9}$

**Table 3:** “GO Biological Process 2018” category of Enrichr for 52 genes selected by applying PCA-based unsupervised FE to gene expression profiles. Six glucose-related terms with adjusted *P*-values less than 0.05 are listed.

Term	Overlap	*P*-value	Adjusted *P*-value
Canonical glycolysis (GO:0061621)	7/25	$2.45\times 10^{-13}$	$6.57\times 10^{-11}$
Glycolytic process through glucose-6-phosphate (GO:0061620)	7/25	$2.45\times 10^{-13}$	$6.93\times 10^{-11}$
Glucose catabolic process to pyruvate (GO:0061718)	7/25	$2.45\times 10^{-13}$	$6.24\times 10^{-11}$
Gluconeogenesis (GO:0006094)	6/41	$9.62\times 10^{-10}$	$2.04\times 10^{-7}$
Glycolytic process (GO:0006096)	5/23	$3.17\times 10^{-9}$	$6.22\times 10^{-7}$
Glucose metabolic process (GO:0006006)	6/64	$1.53\times 10^{-8}$	$2.79\times 10^{-6}$

### TD-based unsupervised FE applied to m6A profiles

**Figure 3:**
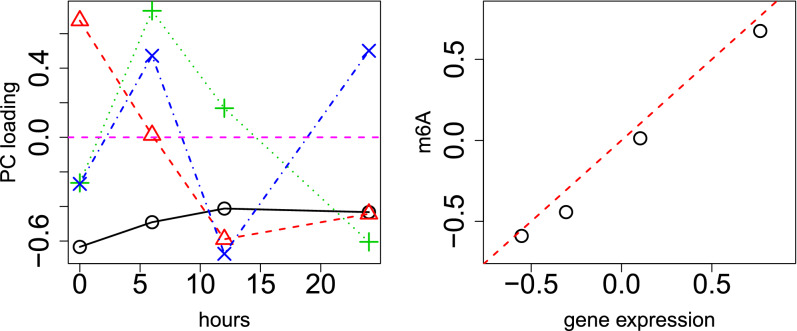
Left: Singular value vector, $u_{\ell _2 t}$, computed by HOSVD applied to time points with applying HOSVD to m6A profiles. Open black circles: 1st (0.78). Open red triangles: 2nd (− 0.80), green crosses: 3rd (− 0.47), blue crosses: 4th PC loading (0.36). The numbers in parentheses are the Pearson’s correlation coefficients. Hours (horizontal axis) represent the duration after the treatments. Horizontal magenta broken line indicates baseline (zero). Right: scatter plot between $v_{2t}$, which is the 2nd PC loading attributed to time points when PCA was applied to $x_{it}$, (horizontal axis) and $u_{2t}$ (vertical axis). The red broken line indicates $v_{2t} = u_{2t}$.

Although we successfully applied PCA-based unsupervised FE to some gene expression profiles of hypoxia, the identification of the relationship between altered gene expression and hypoxia was not the primary purpose of this study. Instead, its purpose was to identify the relationship between m6A profiles and hypoxia. To identify the relationship between hypoxia and m6A profiles, HOSVD was applied to $x_{ktj}$, as described in “Materials and methods”. The left panel in Fig. 3 shows the singular value vectors attributed to time points; the second singular value vector is most significantly correlated with time. Remarkably, $u_{2t}$ is almost identical to $v_{2t}$, which is the 2nd PC loading attributed to the time points when PCA was applied to $x_{it}$ (right panel in Fig. 3). Considering that gene expression and m6A profiles are distinct from each other, this coincidence between $u_{2t}$, which is attributed to m6A profiles, and $v_{2t}$, which is attributed to gene expression profiles, suggests that our analysis correctly detects the regulatory relationship between m6A and the gene expression profile. In addition to the fact that $u_{2t}$ is most significantly correlated with time points, $u_{2j}$s have opposite signs between $j=1$ and $j=2$ (not shown here), which means that $\ell _3=2$ is associated with the distinction between the input and m6A. We then determined which $G(\ell _1,2,2)$ has the largest absolute value to determine which $u_{\ell _1 k}$ is used to select genomic regions, *k* (Table 4). Because it is obvious that *G*(2, 2, 2) has the largest absolute value, we decided to employ $u_{2k}$ to select genomic regions. $P_k$s are attributed to *k* using Eq. (9) with $\ell _1=2$. Then 106 *k*s (genomic regions 25,000 nucleotides in length, see “Materials and methods”) associated with corrected *P*-values less than 0.01, were selected. These 106 genomic regions included 196 unique gene symbols that were uploaded to Enrichr to evaluate enrichment.

**Table 4:** $G(\ell _1,2,2)$ computed by applying HOSVD to $x_{ktj}$.

$\ell _1$	$G(\ell _1,2,2)$
1	$-6.28 \times 10^4$
2	$5.89 \times 10^5$
3	$-5.47 \times 10^4$
4	$9.27 \times 10^4$
5	$-2.24 \times 10^5$
6	$7.59 \times 10^4$
7	$-6.23 \times 10^4$
8	$3.40 \times 10^4$
9	0.0
10	$-1.15 \times 10^{-14}$

In contrast to the 52 genes identified by PCA-based unsupervised FE applied to gene expression, no KEGG pathway terms or GO BP terms were enriched in these 196 gene symbols. Nevertheless, there are some hypoxia experiments in which genes with altered expression are enriched in 196 gene symbols (Table 5). Therefore, even though 196 genes are less biologically significant than the 52 genes identified in the gene expression analysis, they still have some potential to be related to hypoxia.

**Table 5:** “Disease Perturbations from GEO down/up” category of Enrichr for 196 genes selected by applying TD-based unsupervised FE to m6A profiles. Six hypoxia experiments with adjusted *P*-values less than 0.05 are listed.

Term	Overlap	*P*-value	Adjusted * P*-value
**Disease perturbations from GEO down**			
Hypoxia C0242184 mouse GSE3195 sample 70	12/328	$9,99 \times 10^{-5}$	$9,86 \times 10^{-4}$
Hypoxia C0242184 human GSE4483 sample 440	10/275	$3.98 \times 10^{-4}$	$2.79 \times 10^{-3}$
Hypoxia C0242184 human GSE4630 sample 250	10/349	$2.41 \times 10^{-3}$	$1.22 \times 10^{-2}$
**Disease perturbations from GEO up**			
Hypoxia C0242184 human GSE4483 sample 440	22/325	$1.17 \times 10^{-12}$	$4.92 \times 10^{-10}$
Hypoxia C0242184 human GSE4630 sample 250	15/251	$2.82 \times 10^{-8}$	$1.18\times 10^{-6}$
Hypoxia C0242184 mouse GSE3195 sample 70	14/272	$5.15 \times 10^{-7}$	$9.39 \times 10^{-6}$

### Integrated analysis of gene expression and m6A profiles using KTD-based unsupervised FE

**Figure 4:**
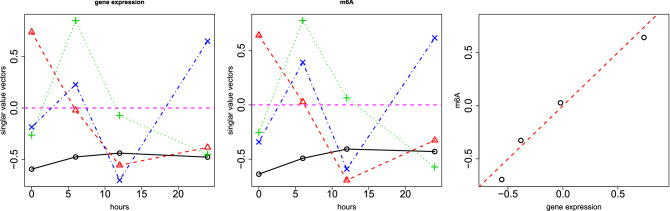
Left: Singular value vector, $u_{\ell _1 t}$, computed by HOSVD applied to $x_{tjt'j'}$. Open black circles: 1st, (0.61) open red triangles: 2nd (− 0.77), green crosses: 3rd (− 0.41), blue crosses: 4th PC loading (0.48). The numbers in parentheses are the Pearson’s correlation coefficients. Hours (horizontal axis) represent the duration after the treatments. Horizontal magenta broken line indicates baseline (zero). Middle: singular value vector, $u_{ \ell _3 t'}$, computed by HOSVD applied to $x_{tjt'j'}$. Open black circles: 1st (0.78). open red triangles: 2nd (− 0.70), green crosses: 3rd (− 0.47), blue crosses: 4th PC loading (0.52). The numbers in parentheses are the Pearson’s correlation coefficients. Hours (horizontal axis) represent the duration after the treatments. Horizontal magenta broken line indicates baseline (zero). Right: scatter plot between $u_{2t}$ (horizontal axis) and $u_{2t'}$ (vertical axis). The red broken line indicates $u_{2t} = u_{2t'}$.

Since TD-based unsupervised FE applied to m6A profiles was not fully successful, we needed to employ more advanced methodology: Kernel TD-based (KTD) unsupervised FE. HOSVD was applied to $x_{tjt'j'}$, as described in “Materials and methods”. Figure 4 shows the results that are consistent with the results obtained by non-integrated analysis (Figs. 2, 3). The second singular value vectors, $u_{2t}$ and $u_{2t'}$, are most consistent with time points; it is coincident with the second PC loading attributed to gene expression, and the second singular value vectors attributed to m6A are coincident with time points. $u_{2t}$ and $u_{2t'}$ are also identical; it is coincident with the second PC loading attributed to gene expression, and the second singular value vectors attributed to m6A are identical. In addition, $u_{2j}$ has the opposite sign between $j=1$ (control) and $j=2$ (m6A). Then $u_{\ell _1 i}$ was computed using Eq. (16), with $\ell _1=2$, and $u_{\ell _2 \ell _3 k}$ was computed using Eq. (17), with $\ell _2=\ell _3=2$. $P_i$ and $P_k$ are attributed to *i* and *k*, respectively, with Eqs. (18) and (19), respectively. The 53 *i*s (genes) and 128 *k*s (genome regions) associated with adjusted *P*-values less than 0.01 were selected. Two hundred gene symbols were retrieved from 128 genomic regions, as previously described.

**Table 6:** “KEGG 2019 Human” category of Enrichr for 53 genes (based on gene expression) and 200 genes (based on m6A) selected by applying KTD-based unsupervised FE to integration of gene expression and m6A profile. Fifteen terms with adjusted *P*-values less than 0.05 are listed.

Term	Overlap	*P*-value	Adjusted *P*-value
**Gene expression**			
Ribosome	20/153	$2.13\times 10^{-29}$	$6.55\times 10^{-27}$
Thermogenesis	13/231	$2.59\times 10^{-14}$	$3.99\times 10^{-12}$
Oxidative phosphorylation	11/133	$4.44\times 10^{-14}$	$4.56\times 10^{-12}$
Parkinson disease	11/142	$9.22\times 10^{-14}$	$7.10\times 10^{-12}$
Glycolysis/gluconeogenesis	8/68	$9.16\times 10^{-12}$	$5.64\times 10^{-10}$
HIF-1 signaling pathway	6/100	$2.55\times 10^{-7}$	$1.31\times 10^{-5}$
Alzheimer disease	7/171	$3.27\times 10^{-7}$	$1.44\times 10^{-5}$
Huntington disease	6/193	$1.18\times 10^{-5}$	$4.53\times 10^{-4}$
Retrograde endocannabinoid signaling	5/148	$4.47\times 10^{-5}$	$1.53\times 10^{-3}$
Cardiac muscle contraction	4/78	$5.42\times 10^{-5}$	$1.67\times 10^{-3}$
Non-alcoholic fatty liver disease (NAFLD)	4/149	$6.52\times 10^{-4}$	$1.83\times 10^{-2}$
**m6A**			
Glycolysis/gluconeogenesis	7/68	$5.21\times 10^{-6}$	$1.60\times 10^{-3}$
Central carbon metabolism in cancer	6/65	$4.69\times 10^{-5}$	$7.22\times 10^{-3}$
HIF-1 signaling pathway	6/100	$5.07\times 10^{-4}$	$5.21\times 10^{-2}$
Glucagon signaling pathway	6/103	$5.93\times 10^{-4}$	$4.57\times 10^{-2}$

The 53 and 200 gene symbols were uploaded to Enrichr. Table 6 shows the results of the “KEGG 2019 Human” category in Enrichr. When compared with Table 1, the enrichment for gene expression profiles is similar. Four enrichment terms were identified, whereas no terms were identified when TD-based unsupervised FE was applied to the m6A profile.

**Table 7:** “Disease Perturbations from GEO down/up” category of Enrichr for 53 genes (based on gene expression) and 200 genes (based on m6A) selected by applying KTD-based unsupervised FE to integration of gene expression and m6A profiles. Nine hypoxia experiments with adjusted *P*-values less than 0.05 are listed.

Term	Overlap	*P*-value	Adjusted *P*-value
**Gene expression**			
**Disease perturbations from GEO down**			
Hypoxia C0242184 human GSE4630 sample 250	15/349	$1.08\times 10^{-14}$	$7.85\times 10^{-14}$
Hypoxia C0242184 mouse GSE3195 sample 70	12/328	$4.53\times 10^{-11}$	$2.03\times 10^{-10}$
Hypoxia C0242184 human GSE4483 sample 440	10/275	$2.38\times 10^{-9}$	$8.67\times 10^{-9}$
**Disease perturbations from GEO up**			
Hypoxia C0242184 human GSE4483 sample 440	27/325	$1.10\times 10^{-34}$	$1.15\times 10^{-32}$
Hypoxia C0242184 mouse GSE3195 sample 70	10/272	$2.14\times 10^{-9}$	$5.79\times 10^{-9}$
Hypoxia C0242184 human GSE4630 sample 250	9/251	$1.83\times 10^{-8}$	$4.51\times 10^{-8}$
**m6A**			
**Disease perturbations from GEO up**			
Hypoxia C0242184 human GSE4483 sample 440	30/325	$1.91\times 10^{-20}$	$1.60\times 10^{-17}$
Hypoxia C0242184 mouse GSE3195 sample 70	21/272	$4.63\times 10^{-13}$	$1.94\times 10^{-10}$
Hypoxia C0242184 human GSE4630 sample 250	17/251	$6.56\times 10^{-10}$	$4.24\times 10^{-8}$

**Table 8:** “GO Biological Process 2018” category of Enrichr for 53 genes (based on gene expression) and 200 genes (based on m6A) selected by applying KTD-based unsupervised FE to integration of gene expression and m6A profiles. Nine glucose-related terms with adjusted *P*-values less than 0.05 are listed.

Term	Overlap	*P*-value	Adjusted *P*-value
**Gene expression**			
Glycolytic process through glucose-6-phosphate (GO:0061620)	7/25	$2.82\times 10^{-13}$	$7.56\times 10^{-11}$
Glucose catabolic process to pyruvate (GO:0061718)	7/25	$2.82\times 10^{-13}$	$6.84\times 10^{-11}$
Canonical glycolysis (GO:0061621)	7/25	$2.82\times 10^{-13}$	$7.18\times 10^{-11}$
Gluconeogenesis (GO:0006094)	6/41	$1.08\times 10^{-9}$	$2.40\times 10^{-7}$
Glycolytic process (GO:0006096)	5/23	$3.49\times 10^{-9}$	$7.13\times 10^{-7}$
Glucose metabolic process (GO:0006006)	6/64	$1.72\times 10^{-8}$	$3.14\times 10^{-6}$
**m6A**			
Glycolytic process through glucose-6-phosphate (GO:0061620)	5/25	$4.29\times 10^{-6}$	$2.19\times 10^{-2}$
Glucose catabolic process to pyruvate (GO:0061718)	5/25	$4.29\times 10^{-6}$	$7.30\times 10^{-3}$
Canonical glycolysis (GO:0061621)	5/25	$4.29\times 10^{-6}$	$1.10\times 10^{-2}$

Table 7 shows the results of the “Disease Perturbations from GEO down/up” category in Enrichr. Compared with Tables 2 and 5, although enrichment in the “Disease Perturbations from GEO down” for m6A is missing, it still has enrichment for both gene expression and m6A profiles. Table 8 shows the enrichment in the “GO Biological Process 2018” category of Enrichr. Compared with Table 3, enrichment for gene expression does not change, and enrichment for m6A is identified, whereas it was not identified when TD-based unsupervised FE was applied to the m6A profile. Thus, KTD-based unsupervised FE improved the enrichment of m6A profiles without affecting the enrichment for gene expression.

**Table 9:** Confusion matrices of selected genes between gene expression and m6A profiles. PCA-and TD-based unsupervised FE were separately applied to gene expression and m6A profiles, or KTD-based unsupervised FE was applied to the integration of gene expression and m6A profiles

		PCA-and TD-based unsupervised FE		KTD-based unsupervised FE	
m6A	Gene expression	Not selected	Selected	Not selected	Selected
	Not selected	19773	45	19783	41
	Selected	189	7	188	12
	Odds ratio	16.26		30.77	
	*P*-value	$7.15 \times 10^{-7}$		$1.32 \times 10^{-13}$	

Additional improvement from PCA- and TD-based unsupervised FE to integrated analysis using KTD-based unsupervised FE identified significant associations between gene expression and m6A (Table 9). When PCA-and TD-based unsupervised FE were separately applied to gene expression and m6A profiles, 52 and 196 genes were identified, respectively. The number of common genes between them was seven. However, integrated analysis of gene expression and m6A profiles with TKD-based unsupervised FE identified 53 genes for gene expression and 200 genes for m6A profiles. The number of common genes increased to 12. Although we cannot estimate their significance very accurately, if we can tentatively assume that there are 20,000 human genes in total, both coincidences are significant, and coincidence in KTD-based unsupervised FE is more significant.

In conclusion, integrated analysis of gene expression and m6A profiles using KTD-based unsupervised FE substantially increased the results compared with applying PCA- and TD-based unsupervised FE separately to gene expression and m6A profiles, respectively. KTD-based unsupervised FE could identify the relationship of gene expression and m6A with hypoxia simultaneously for the first time in a statistically significant manner.

### Comparisons with other conventional methods

Although we have shown that integrated analysis of gene expression and m6A profiles simultaneously identified the relationship between hypoxia and gene expression as well as hypoxia and m6A profiles, if other simpler conventional methods can achieve similar performances, it is useless to employ complicated methods such as KTD-based unsupervised FE. To confirm that other conventional methods cannot identify similar relationships, we applied a few conventional feature selection methods. As can be seen in the following text, no conventional feature selections were found to be useful.

#### Linear regression analysis

Linear regressions were applied to the gene expression profiles, $x_{it}$, and m6A profiles, $x_{ktj}$. No genes or genomic regions was associated with adjusted *P*-values less than 0.05, respectively; thus, no genes or genomic regions were associated with adjusted *P*-values less than 0.01 either.

#### SAM

Although we tried to apply SAM to gene expression profiles, $x_{it}$, and m6A profiles, $x_{ktj}$, we found that SAM requires at least two replicates for each class. In this study, there are no replicated classes in four classes in $x_{it}$ or eight classes in $x_{ktj}$; therefore, we could not apply SAM to these data sets.

#### Limma

Limma was applied to the gene expression profiles, $x_{it}$, and m6A profiles, $x_{ktj}$. No genes but 72252 genomic regions were associated with adjusted *P*-values of less than 0.01, respectively. Therefore, limma was not useful.

#### Random forest

Random forest was applied to gene expression profiles, $x_{it}$, and m6A profiles, $x_{ktj}$. Four hundred and eighty genes and 722 genomic regions had non-zero importance, respectively. Thus, random forest successfully selected a reasonable number of genes and genomic regions. Nevertheless, no hypoxia-related biological terms were enriched in the 480 genes or gene symbols included in the 722 genomic regions. Thus, random forest was not a useful method.

## Discussion

One might wonder why linear regression, SAM, limma, and random forest failed to select genes associated with altered gene expression, genomic regions associated with m6A profiles in hypoxia, or genes biologically related to hypoxia. This is because it is a very difficult problem. There are more than 17140 genes as well as 123817 genomic regions, whereas the number of samples measured was four and eight, respectively, which were too small to obtain sufficiently significant *P*-values. These numbers of genes and genomic regions were too large to obtain significant *P*-values; although random forest is free from *P*-values, too small sample numbers often prevent random forest from obtaining results that are not obtainable by chance.

To demonstrate how the KTD-based unsupervised FE outperforms the other four methods, we applied them to two synthetic data sets with $N=1000$ and $N_1=10$. When linear regression was applied to the 1st synthetic data set, there were no *i*s associated with adjusted *P*-values less than 0.01. When limma and random forest were applied to the 1st synthetic data set, there were as many as 500 *i*s associated with adjusted *P*-values less than 0.01. Thus, neither linear regression nor limma was useful.

The problem is when time points are regarded as a quantitative property (i.e., in linear regression); in this case, eight samples were too small to give significant *P*-values because there are 1000 features on which *P*-values must be corrected by considering multiple comparison criteria. However, if they are classified into eight classes with one replicate, too many *i*s were regarded as distinct between eight classes because those values that are constant between any pairs of eight classes are unlikely to be fulfilled when using a null hypothesis.

**Figure 5:**
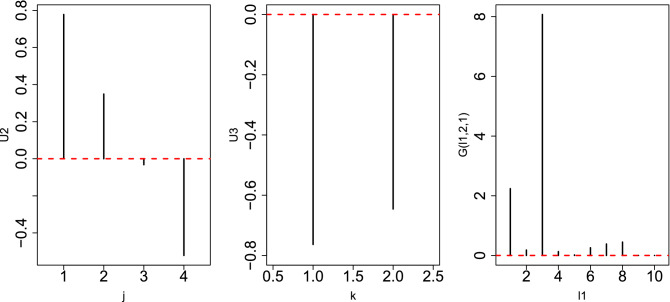
Left: Singular value vector, $u_{2 j}$, computed by HOSVD applied to the 1st synthetic data, $x_{ijk}$. Middle: Singular value vector, $u_{1 k}$, computed by HOSVD applied to 1st synthetic data, $x_{ijk}$. Right: $|G(\ell _1,2,1)|$. In all three sub-panels, the horizontal red broken lines indicate the baseline (zero).

**Figure 6:**
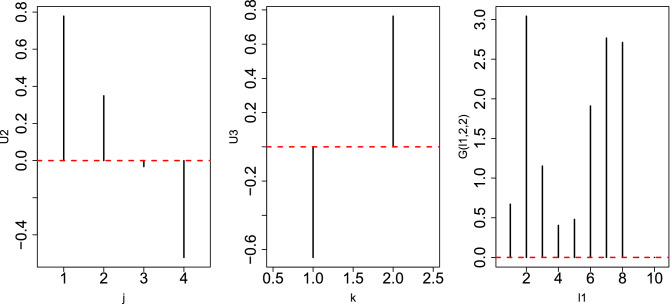
Left: Singular value vector, $u_{2 j}$, computed by HOSVD applied to the 1st synthetic data, $x_{ijk}$. Middle: singular value vector, $u_{2 k}$, computed by HOSVD applied to 1st synthetic data, $x_{ijk}$. Right: $|G(\ell _1,2,2)|$. In all three sub-panels, the horizontal red broken lines indicate the baseline (zero).

**Figure 7:**
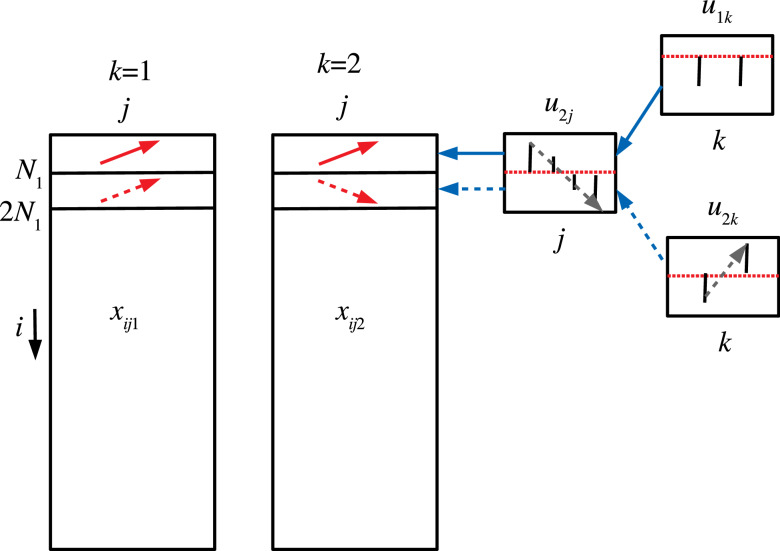
Schematic figure that explains the correspondence between $x_{ijk}, i \le N_1$ (red solid arrows) and $u_{2j}$, $u_{1k}$ and that between $x_{ijk}, N_1< i \le 2 N_1$ (red broken arrows) and $u_{2j}$ and $u_{2k}$, respectively.

However, when TD-based unsupervised FE was applied to the first synthetic data set, the situation was very different. Figures 5 and 6 show the two combinations of the $u_{\ell _2,j}$, $u_{\ell _3 k}$, and $G(\ell _1 \ell _2 \ell _3)$. Figure 5 ($\ell _2 =2, \ell _3=1$) corresponds to $x_{ijk}, i \le N_1$ since $x_{ij1} =x_{ij2}$ is coincident with $u_{\ell _3 1} = u_{\ell _3 2}$ (see Fig. 7). However, Fig. 6 ($\ell _2 = \ell _3=2$) corresponds to $x_{ijk}, N_1 < i \le 2 N_1$ because $x_{ij1} = - x_{ij2}$, which coincides with $u_{\ell _3 1} = - u_{\ell _3 2}$ (see Fig. 7). Thus, in contrast to other supervised methods, TD-based unsupervised FE can detect the distinction between $x_{ij1}= x_{ij2}$ and $x_{ij1} = - x_{ij2}$. To determine whether TD-based unsupervised FE can correctly identify $x_{ijk}$s, we need to attribute *P*-values to *i*s. Because $|G(\ell _1,2,1)|$ and $|G(\ell _1,2,2)|$ have the largest values when $\ell _1=3$ and $\ell _1=2$, respectively, we decided to attribute *P*-values to *i*s using Eq. (13) by assigning $\ell _1=3$ and $\ell _1=2$, respectively. Computed *P*-values were corrected, and *i*s associated with adjusted *P*- values less than 0.01 were selected. Table 10 shows the performance of TD-based unsupervised FE applied to the first synthetic data set. It perfectly selects *i* coincident with Eq. (1). As a result, the reason why only PCA and TD-based unsupervised FE could select a reasonable number of genes, while other methods failed, is because PCA and TD-based unsupervised FE are suitable for situations where there are a very small number of samples with a large number of features (observations).

**Table 10:** Confusion matrices of selected is between true and those selected by TD-based unsupervised FE applied to synthetic data set 1.

TD based unsupervised FE	$\ell _1=3$			$\ell _1=2$	
Adjusted *P*-values	$>0.01$	$\le 0.01$		$>0.01$	$\le 0.01$
$i \not \in [1,N_1]$	990	0	$i \not \in [N_1+1,2N_1]$	990	0
$i \in [1,N_1]$	0	10	$i \in [N_1+1,2N_1]$	0	10

**Figure 8:**
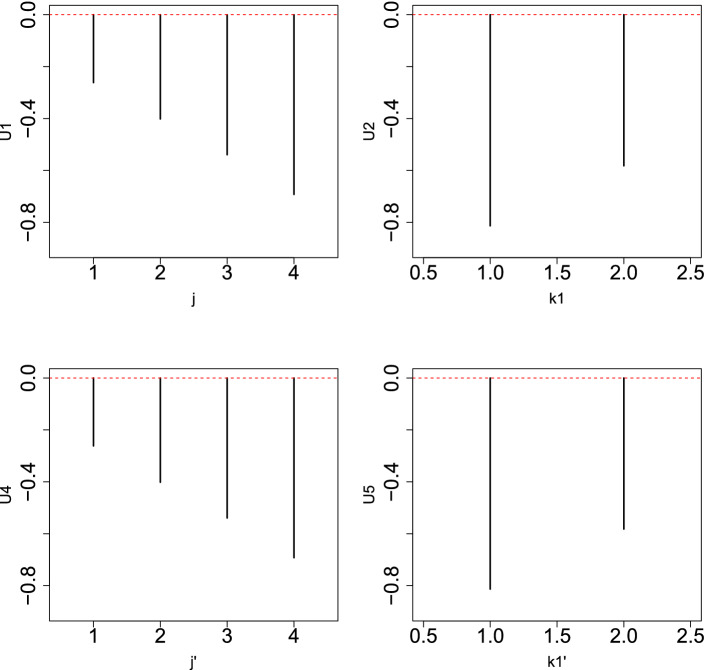
Singular value vectors obtained when KTD-based unsupervised FE was applied to the 2nd synthetic data set. Upper left: $u_{1 j}$, upper right: $u_{1 k_1}$, lower left: $u_{1 j'}$, lower right: $u_{1 k'_1}$. The horizontal red broken line indicates baseline (zero).

**Figure 9:**
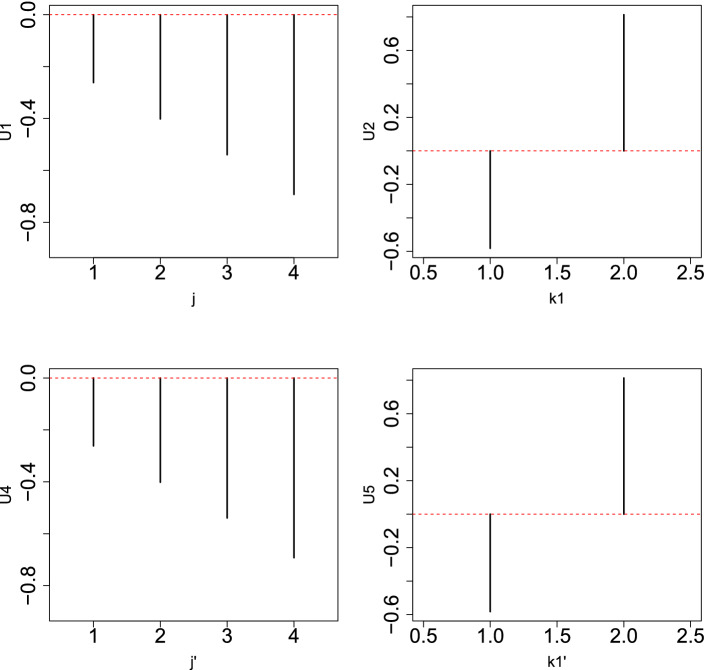
Singular value vectors obtained when KTD-based unsupervised FE was applied to the 2nd synthetic data set. Upper left: $u_{1 j}$, upper right: $u_{2 k_1}$, lower left: $u_{1 j'}$, lower right: $u_{2 k'_1}$. The horizontal red broken line indicates baseline (zero).

Next, we attempted to demonstrate how KTD-based unsupervised FE integrates two data sets in order to identify common features between the two. Figures 8 and 9 show singular value vectors obtained when KTD-based unsupervised FE was applied to $x_{jk_1k_2j'k'_1k'_2}$.

**Figure 10:**
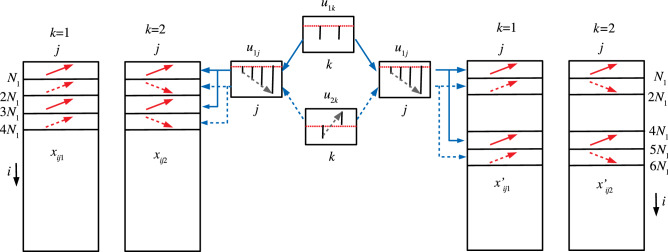
Schematic figure that explains the correspondence between $x_{ijk}, i \le N_1, 2N_1< i \le 3N_1$, $x'_{ijk}, i \le N_1, \; {\text {and}} \; 4N_1< i \le 5N_1$ (red solid arrows) and $u_{1j}$ and $u_{1k}$ and that between $x_{ijk}, N_1< i \le 2 N_1, 3N_1 < i \le 4N_1$, $x'_{ijk}, N_1< i \le 2 N_1, \; {\text {and}} \; 5N_1 < i \le 6N_1$ (red broken arrows) and $u_{1j}$ and $u_{2k}$, respectively.

Figure 8 shows $u_{1 j}, u_{1 k_1}, u_{1 j'}$, and $u_{1 k'_1}$, which is coincident with $x_{ijk}, i \le N_1$, and $2N_1 <i \le 3N_1$ and $x'_{ijk}, i \le N_1$, and $4N_1 < i \le 5N_1$, since both singular value vectors and $x_{ijk}$ and $x'_{ijk}$ for these *i*s increase as *j* increases and do not depend on *k* (see Fig. 10). To determine whether we can select these $x_{ijk}$ and $x'_{ijk}$ in these *i*s, we reproduced singular value vectors attributed to *i*s using Eq. (22) with $\ell _1=\ell _2=1$ or Eq. (23) with $\ell _4=\ell _5=1$. Then *P*-values were attributed to *i*s using Eq. (24) or Eq.   (25). Computed *P*-values were corrected, and *i*s associated with adjusted *P*-values less than 0.01 were selected. Table 11 shows a perfect performance.

**Table 11:** Confusion matrices of selected is between true and those selected by KTD-based unsupervised FE applied to synthetic data set 2.

KTD based unsupervised FE	$\ell _1=\ell _2=1$			$\ell _4=\ell _5=1$	
Adjusted *P*-values	$>0.01$	$\le 0.01$		$>0.01$	$\le 0.01$
$i \not \in [1,N_1] \cup [2 N_1+1, 3N_1]$	980	0	$i \not \in [1,N_1] \cup [4 N_1+1, 5N_1]$	980	0
$i \in [1,N_1] \cup [2 N_1+1, 3N_1]$	0	20	$i \in [1,N_1] \cup [4 N_1+1, 5N_1]$	0	20

	$\ell _1=1,\ell _2=2$			$\ell _4=1,\ell _5=2$	
Adjusted *P*-values	$>0.01$	$\le 0.01$		$>0.01$	$\le 0.01$
$i \not \in [N_1+1,2N_1] \cup [3 N_1+1, 4N_1]$	980	0	$i \not \in [N_1+1,2N_1] \cup [5 N_1+1, 6N_1]$	980	0
$i \in [N_1+1,2N_1] \cup [3 N_1+1, 4N_1]$	0	20	$i \in [N_1+1,2N_1] \cup [5 N_1+1, 6N_1]$	0	20

Figure 9 shows $u_{1 j}, u_{2 k_1}, u_{1 j'}$, and $u_{2 k'_1}$, which is coincident with $x_{ijk}, N_1 < i \le 2N_1$, and $3N_1 <i \le 4N_1$, and $x'_{ijk}, N_1 < i \le 2N_1$, and $5N_1 < i \le 6N_1$, since $u_{1 j}$ and $u_{1 j'}$ increase as *j* increases, and $u_{2 k_1}$ and $u_{2 k'_1}$ have opposite signs between $k_1=k'_1=1$ and $k_2=k'_2=2$, while $x_{ij1}$ and $x'_{ijk1}$ for these *i*s increase as *j* increases, and $x_{ij2}$ and $x'_{ij2}$ for these *i*s decrease as *j* increases (see Fig. 10). To determine whether we can select these $x_{ijk}$ and $x'_{ijk}$ in these *i*s, we reproduced singular value vectors attributed to *i*s using Eq. (22) with $\ell _1=1, \ell _2=2$ or Eq. (23) with $\ell _4=1, \ell _5=2$. Then, *P*-values were attributed to *i*s using Eq. (24) or Eq.   (25). Computed *P*-values were corrected, and *i*s associated with adjusted *P*-values less than 0.01 were selected. Table 11 shows a perfect performance.

**Table 12:** Confusion matrix of selected *i*s between $x_{ijk}$ and $x'_{ijk}$ by KTD-based unsupervised FE applied to synthetic data set 2.

		$$x'_{ijk}$$	
$x_{ijk}$	Adjusted *P*-values	$>0.01$	$\le 0.01$
	$>0.01$	970	10
	$\le 0.01$	10	10

Table 12 shows the confusion matrix between *i*s selected for $x_{ijk}$ and those selected for $x'_{ijk}$. This corresponds to Table 9, where genes were selected based on gene expression and m6A profiles. This might be the reason why KTD-based unsupervised FE could identify a significantly overlapping set of genes between gene expression and m6A profiles.

Although TD- and KTD-based unsupervised FE can outperform conventional supervised methods when applied to a small number of samples with a large number of features, TD- and KTD-based unsupervised FE have yet another advantage: *j* dependence is not monotonic (see the open red triangles in Figs. 2, 3, and 4). Such a non-linear dependence on *j* cannot be assumed by supervised methods in advance. Wrongly assumed *j* dependence results in decreased feature selection performance. This is another reason why PCA-, TD-, and KTD-based unsupervised FE can outperform other conventional supervised feature selection methods.

In order to see if our findings are robust, we tried to find alternative data sets in which gene expression and m6A were simultaneously measured for hypoxia, but we could not find any such data sets. Thus, we employed GSE120860, in which only m6A was measured. TD-based unsupervised FE applied to these data sets gave us 54 genes associated with adjusted *P*-values less than 0.01 ($\ell _2=\ell _3=1$ and $\ell _4=2$ were selected, and $u_{4 i}$ was used to attribute *P*-values to gene *i* with Eq. (11) because *G*(4, 1, 1, 2) has the largest absolute value given $\ell _2=\ell _3=1$ and $\ell _4=2$). Uploading these genes to Enrichr did not identify any terms associated with both hypoxia and significant *P*-values. As shown in Table 5, when genes were selected by m6A only, there were fewer significant terms. Therefore, we may need to have alternative data sets associated with both gene expression and m6A simultaneously in order to validate the robustness of our results.

One might wonder why we did not compare the proposed methods with conventional unsupervised methods using PCA and TD but only with supervised methods. The reasons for this are as follows. Although there are many papers whose titles include “Feature selection using principal component analysis”, feature selections in these papers mean selecting limited numbers of latent vectors generated by PCA or TD. Thus, they are not applicable to the present study, which needed to select not generated features but original ones (i.e., genomic regions). Although there are a few studies that aim to select original features, and not generated latent vectors, they did not attribute *P*-values to the features, which would have allowed us to evaluate the significance of the feature selections. For example, Song et al.^24^ selected a limited number of original features associated with relatively larger absolute values of eigenvectors, and no *P*-values were attributed to the individual original features. The purpose of the present study is not simply to select features but to evaluate the significance of selected features; as denoted above, Song et al’s study could not help us to evaluate the significance of the feature selections. This is why we did not compare our method with other unsupervised methods using PCA or TD but compared ours with the supervised methods that could give us *P*-values, by which we could evaluate the significance of the feature selections.

In this study, we applied PCA-, TD-, and KTD-based unsupervised FE to gene expression and m6A profiles in hypoxia. Although these methods identified a limited number of genes significantly related to hypoxia, other conventional methods failed. To understand why PCA-, TD-, and KTD-based unsupervised FE could outperform other conventional methods, we applied these methods to synthetic data sets with small numbers of samples and large numbers of features. As a result, we successfully reproduced the superior performance of TD-and KTD-based unsupervised FE over other conventional methods. Thus, the superiority of PCA-, TD-, and KTD-based unsupervised FE is possibly due to having a small number of samples with a large number of features. In conclusion, despite the limitations of previous studies, we validated a set of genes associated with altered gene expression and m6A profiles in hypoxia in a statistically significant manner.

## Materials and methods

### m6A and gene expression profiles

m6A and gene expression profiles were downloaded from Gene Expression Omnibus (GEO) using GEO ID GSE141941. For m6A, eight files included in GSE141941_RAW.tar available as part of the Supplementary Information were employed. m6A profiles were summed up within 25,000-nucleotide intervals sequentially divided over the whole genome. As a result, 123,817 genomic regions of 25,000 nucleotides in length were obtained. For gene expression, four profiles included in GSE141941_normoxiaVShypoxia6h.12h.24h_RNA-seq.PROCESSED.DATA.xlsx, which is also available as a part of the Supplementary Information, were employed. Eight files for m6A were composed of four time points (including the control), time input, or treated files. Four profiles for gene expression were composed of four times points as well.

As an alternative data set, we employed GSE120860, in which only the m6A profile was measured. We downloaded 16 bed files provided in the Supplementary Information section in GEO, which correspond to four healthy controls and four patients, of which the tumor and paratumor were measured.

### Synthetic data set

In order to demonstrate how well KTD-based unsupervised FE can work when there are a small number of samples associated with a large number of features (observations) and where other conventional supervised methods fail, we prepared a synthetic data set. It has *N* variables attributed to eight samples whose number is the same as that of the m6A profiles. In the first data set, we aimed to demonstrate the performance when KTD-based unsupervised FE is applied to a single data set. It is composed of ${x_{ijk}} \in {{\mathbb {R}}^{N \times 4 \times 2}}$,

1 $$\begin{aligned} x_{ijk} = \left\{ \begin{array}{crc} j + \epsilon _{ijk} &{} i \le N_1, \\ j + \epsilon _{ijk} &{} N_1< i \le 2 N_1, &{} k=1 \\ -j + \epsilon _{ijk}&{} N_1 <i \le 2 N_1, &{} k=2, \end{array} \right. \end{aligned}$$

where *j* corresponds to the first to fourth time points, and $k=1$ and $k=2$ correspond to two distinct experimental conditions. $\epsilon _{ijk}$ obeys ${{\mathcal {N}}}(0,\frac{1}{2})$, where ${{\mathcal {N}}} (\mu , \sigma )$ is a Gaussian distribution with a mean of $\mu$ and a standard deviation of $\sigma$. For $i \le N_1$, the two conditions have the same dependence on time points, whereas for $N_1 < j \le 2 N_2$, the two conditions have opposite time point dependence.

In the second dataset, we aimed to demonstrate how KTD-based unsupervised FE can identify features that share the same time point dependence on two measurements represented as two tensors, ${x_{ijk}} \; \mbox{and} \; {x'_{ijk}} \in {{\mathbb {R}}^{N \times 4 \times 2}}$, which obey Eq. (1) for $i \le 2 N_1$, and for $i > 2 N_1$,

2 $$\begin{aligned} x_{(i+2N_1) jk}= & {} x_{ijk} , i \le 2 N_1 \end{aligned}$$

3 $$\begin{aligned} x'_{(i+4N_1)jk}= & {} x_{ijk} , i \le 2 N_1. \end{aligned}$$

Thus, for $i \le 2 N_1$, $x_{ijk}$, $x_{(i+2N_1) jk}$, $x'_{ijk}$, and $x'_{(i+4N_1)jk}$ share the same time-point dependence.

### PCA-based unsupervised FE applied to gene expression

Although the details of PCA-based unsupervised FE have been described in a recently published book^25^, we briefly outline this method. Suppose we have gene expression profiles as a matrix, ${x_{it}} \in {{\mathbb {R}}^{N \times 4}}$, which represents the gene expression of the *i*th gene at the *t*th time point. The $\ell$th PC score attributed to gene *i*, ${u_{\ell i}} \in {{\mathbb {R}}^N}$, can be obtained as the *i*th component of the $\ell$th eigenvector, $\varvec{u}_\ell$, of the gram matrix $X X^T \in {{\mathbb {R}}^{N \times N}}$, where *X* is an $N \times 4$ matrix composed of $x_{it}$, and $X^T$ is a transposed matrix of *X* as

4 $$\begin{aligned} XX^T \varvec{u}_\ell = \lambda _\ell \varvec{u}_\ell . \end{aligned}$$

In contrast, the PC loading attributed to the *t*th time point can be computed as the *t*th component of the $\ell$th PC loading vector, $\varvec{v}_\ell \in {{\mathbb {R}}^4}$, which can be computed as

5 $$\begin{aligned} \varvec{v}_\ell = X^T \varvec{u}_\ell . \end{aligned}$$

This is also the $\ell$th eigenvector of $X^T X \in {{\mathbb {R}}^{4 \times 4}}$ because

6 $$\begin{aligned} X^T X \varvec{v}_\ell = X^T X X^T \varvec{u}_\ell = X^T \lambda _\ell \varvec{u}_\ell = \lambda _\ell X^T \varvec{u}_\ell = \lambda _\ell \varvec{v}_\ell . \end{aligned}$$

In order to select genes, we first need to determine which $\varvec{v}_\ell$ is associated with time dependence. After identifying the time-dependent $\varvec{v}_\ell$, we attribute *P*-values to the *i*th gene using $u_{\ell i}$ by assuming that $u_{\ell i}$ follows a Gaussian distribution (null hypothesis)

7 $$\begin{aligned} P_i = P_{\chi ^2} \left[ > \left( \frac{u_{\ell i}}{\sigma _\ell }\right) ^2 \right] , \end{aligned}$$

where $P_{\chi }[>x]$ is the cumulative $\chi ^2$ distribution in which the argument is larger than *x*, and $\sigma _\ell$ is the standard deviation. The computed *P*-values were corrected by the BH criterion^25^, and genes associated with adjusted *P*-values less than 0.01 were selected.

### TD-based unsupervised FE applied to the m6A profile

Although the details of TD-based unsupervised FE are described in a recently published book^25^, we briefly outline this method. For GSE141941, suppose that a tensor $x_{ktj} \in {{\mathbb {R}}^{K \times 4 \times 2}}$ represents the m6A of the *k*th genomic region at the *t*th time point of input (control, $j=1$) or m6A ($j=2$) sample. Individual genomic regions are 25,000-nucleotide sequence length regions sequentially defined over the whole genome without overlaps and adjusted with each other. Higher-order singular value decomposition^25^ (HOSVD) was applied to $x_{ktj}$, and TD was obtained as

8 $$\begin{aligned} x_{ktj} = \sum _{\ell =1}^{K} \sum _{\ell _2=1}^4 \sum _{\ell _3=1}^2 G(\ell _1 \ell _2 \ell _3) u_{\ell _1 k } u_{\ell _2 t} u_{\ell _3 j }, \end{aligned}$$

where $G \in {{\mathbb {R}}^{K \times 4 \times 2}}$ is the core tensor, $u_{\ell _1 k} \in {\mathbb {R}}^{K \times K}$, $u_{\ell _2 t} \in {\mathbb {R}}^{4 \times 4}$, and $u_{\ell _3 j} \in {\mathbb {R}}^{2 \times 2}$ are singular value matrices and orthogonal matrices.

To select genomic regions that are associated with time dependence and to distinguish between input and m6A treatment, we need to specify which $u_{\ell _2 t}$ and $u_{\ell _3 j}$ are associated with time dependence and the distinction between control (input) and m6a, respectively. Once $u_{\ell _2 t}$ and $u_{\ell _3 j}$ are fixed, we attempt to find $G(\ell _1 \ell _2 \ell _3)$ with the largest absolute value, given $\ell _2$ and $\ell _3$. Finally, we attribute the *P*-value to the *k*th genomic region by assuming that $u_{\ell _1 k}$ obeys a Gaussian distribution (null hypothesis) as

9 $$\begin{aligned} P_k = P_{\chi ^2} \left[ > \left( \frac{u_{\ell _1 k}}{\sigma _{\ell _1}}\right) ^2 \right] , \end{aligned}$$

where $\sigma _{\ell _1}$ is the standard deviation. The computed *P*-values were corrected by the BH criterion, and genes associated with adjusted P-values less than 0.01 were selected.

For GSE126860, m6A profiles were formatted as $x_{ijkm} \in {\mathbb {R}}^{N \times 4 \times 2 \times 2}$, which represents the m6A profiles of the *i*th gene at the *j*th subject of the *k*th group ($k=1$: annotated as 0204 in GEO, $k=2$: annotated as patient in GEO) of the *m*th tissue ($m=1$:tumor, $m=2$:paratumor). HOSVD was applied, and we obtained

10 $$\begin{aligned} x_{ijkm} = \sum _{\ell =1}^{N} \sum _{\ell _2=1}^4 \sum _{\ell _3=1}^2 \sum _{\ell _4=1}^2 G(\ell _1 \ell _2 \ell _3 \ell _4) u_{\ell _1 i } u_{\ell _2 j} u_{\ell _3 k } u_{\ell _3 m }, \end{aligned}$$

where $G \in {\mathbb {R}}^{N \times 4 \times 2 \times 2}$ is the core tensor, and $u_{\ell _1 i} \in {\mathbb {R}}^{N \times N}$, $u_{\ell _2 j} \in {\mathbb {R}}^{4 \times 4}$, and $u_{\ell _3 k}, u_{\ell _4 m},\in {\mathbb {R}}^{2 \times 2}$ are singular value matrices and orthogonal matrices.

In order to select genes that are associated with the distinction between tumor and paratumor, but independent of subjects as well as groups, we need to specify which $u_{\ell _4 m}$ are associated with the distinction between the tumor and paratumor, but $u_{\ell _2 j}$ and $u_{\ell _3 k}$ take constant values. Once $u_{\ell _2 j}, u_{\ell _3 k}, \; \mbox{and} \; u_{\ell _4 m}$ are fixed, we then attempt to find that $G(\ell _1 \ell _2 \ell _3 \ell _4)$ with the largest absolute value, given $\ell _2, \ell _3, \; \mbox{and} \; \ell _4$. Finally, we attribute the *P*-value to the *i*th gene by assuming that $u_{\ell _1 i}$ obeys a Gaussian distribution (null hypothesis) as

11 $$\begin{aligned} P_i = P_{\chi ^2} \left[ > \left( \frac{u_{\ell _1 i}}{\sigma _{\ell _1}}\right) ^2 \right] , \end{aligned}$$

where $\sigma _{\ell _1}$ is the standard deviation. The computed *P*-values were corrected by the BH criterion, and genes associated with adjusted P-values less than 0.01 were selected.

When HOSVD was applied to the 1st synthetic data set, we obtained

12 $$\begin{aligned} x_{ijk} = \sum _{\ell _1=1}^N \sum _{\ell _2=1}^4 \sum _{\ell _3=1}^2 G(\ell _1 \ell _2 \ell _3) u_{\ell _1 i} u_{\ell _2 j} u_{\ell _3 k}, \end{aligned}$$

where $G \in {\mathbb {R}}^{N \times 4 \times 2}$ is the core tensor and $u_{\ell _1 i} \in {\mathbb {R}}^{N \times N}, u_{\ell _2 j} \in {\mathbb {R}}^{4 \times 4}, \; \mbox{and} \; u_{\ell _3 k} \in {\mathbb {R}}^{2 \times 2}$ are singular value matrices and orthogonal matrices.

After identifying $u_{\ell _2 j}$ and $u_{\ell _3 k}$ associated with properties of interest, we attempt to find $G(\ell _1 \ell _2 \ell _3)$ with the largest absolute value, given $\ell _2, \ell _3$. Using the selected $\ell _1$, *P*-values are attributed to the *i*th by assuming that $u_{\ell _1 i}$ obeys a Gaussian distribution,

13 $$\begin{aligned} P_i = P_{\chi ^2} \left[ > \left( \frac{u_{\ell _1 i}}{\sigma _{\ell _1}}\right) \right] . \end{aligned}$$

The computed *P*-values were corrected by the BH criterion, and genes associated with adjusted *P*-values less than 0.01 were selected.

### Integrated analysis of gene expression and m6A profiles

To integrate gene expression and m6A profiles, we employed a recently proposed KTD-based unsupervised FE^26^. We define a tensor $x_{tjt'j'} \in \mathbb{R}^{4 \times 2 \times 4 \times 2}$ as

14 $$\begin{aligned} x_{tjt'j'} = \sum _{t''} \left( \sum _i x_{it}x_{it''} \right) \left( \sum _k x_{kt''j}x_{kt'j'} \right) . \end{aligned}$$

HOSVD was applied to $x_{tjt'j'}$, and we obtained

15 $$\begin{aligned} x_{tjt'j'} = \sum _{\ell _1=1}^4 \sum _{\ell _2=1}^2 \sum _{\ell _3=1}^4 \sum _{\ell _4=1}^2 G(\ell _1 \ell _2 \ell _3 \ell _4) u_{\ell _1 t} u_{\ell _2 j}u_{\ell _3 t'}u_{\ell _4 j'}, \end{aligned}$$

where $G \in {\mathbb {R}}^{4 \times 2 \times 4 \times 2}$ is the core tensor, and $u_{\ell _1 t} \in {\mathbb {R}}^{4 \times 4}$, $u_{\ell _2 t'} \in {\mathbb {R}}^{2 \times 2}$, $u_{\ell _3 j} \in {\mathbb {R}}^{4 \times 4}$, and $u_{\ell _4 j'} \in {\mathbb {R}}^{2 \times 2}$ are singular value matrices and orthogonal matrices. Here, it should be noted that $u_{\ell _2 j}, u_{\ell _3 t'}, \; \mbox{and} \; u_{\ell _4 j'}$ are attributed to m6A profiles, and only $u_{\ell _1 t}$ is attributed to the gene expression profiles.

In order to identify genes whose expression profiles depend on time and genomic regions where m6A profiles depend on time associated with the distinction between m6A and control, we need to find which $u_{\ell _1 t}$ and $u_{\ell _3 t'}$ depend on time and which $u_{\ell _2 j}$ and $u_{\ell _4 j'}$ are distinct between control and m6A (since $x_{tjt'j'}$ does not change even if *j* is replaced with $j'$, $u_{\ell _2 j} = u_{\ell _4 j'}$). Once $\ell _1, \ell _2, \ell _3, \; \mbox{and} \; \ell _4$ are identified, we can compute the singular value vectors attributed to gene expression samples, $u_{\ell _1 i}$, and m6A profiles, $u_{\ell _2 \ell _3 k}$, can be computed as

16 $$\begin{aligned} u_{\ell _1 i}= & {} \sum _{t=1}^4 u_{\ell _1 t} x_{it} \end{aligned}$$

17 $$\begin{aligned} u_{\ell _2 \ell _3 k}= & {} \sum _{j=1}^2 \sum _{t=1}^4 u_{\ell _2 j} u_{\ell _3 t} x_{ktj}. \end{aligned}$$

The *P*-values are attributed to *i*s and *k*s as

18 $$\begin{aligned} P_i= & {} P_{\chi ^2} \left[ > \left( \frac{u_{\ell _1 i}}{\sigma _{\ell _1}}\right) ^2 \right] , \end{aligned}$$

19 $$\begin{aligned} P_k= & {} P_{\chi ^2} \left[ > \left( \frac{u_{\ell _2 \ell _3 k}}{\sigma _{\ell _2 \ell _3}}\right) ^2 \right], \end{aligned}$$

where $\sigma _{\ell _1}$ and $\sigma _{\ell _2 \ell _3}$ are standard deviations. The computed *P*-values were corrected by the BH criterion, and genes, *i*, and genomic regions, *k*, associated with adjusted P-values less than 0.01 were selected.

### Integrated analysis of the 2nd synthetic data set

To integrate $x_{ijk}$ and $x'_{ijk}$ in the second synthetic data set, we define a tensor, $x_{jk_1 k_2 j'k'_1k'_2} \in {\mathbb {R}}^{4 \times 2\times 2 \times 4 \times 2 \times 2}$, as

20 $$\begin{aligned} x_{jk_1 k_2 j'k'_1k'_2}= \sum _{j''} \left( \sum _i x_{ijk_1}x_{ij''k_2} \right) \left( \sum _i x'_{ij''k'_1}x_{ij'k'_2} \right) . \end{aligned}$$

After applying HOSVD to $x_{jk_1 k_2 j'k'_1k'_2}$, we obtained

21 $$\begin{aligned} x_{jk_1 k_2 j'k'_1k'_2}= & {} \sum _{\ell _1=1}^4 \sum _{\ell _2=1}^2 \sum _{\ell _3=1}^2 \sum _{\ell _4=1}^4 \sum _{\ell _5=1}^2 \sum _{\ell _5=1}^2 G(\ell _1 \ell _2 \ell _3 \ell _4 \ell _5 \ell _6) \nonumber \\\times & {} u_{\ell _1 j} u_{\ell _2 k_1}u_{\ell _3 k_2}u_{\ell _4 j'}u_{\ell _5 k'_1}u_{\ell _6 k'_2}. \end{aligned}$$

Singular value vectors attributed to *i*s can be reproduced as

22 $$\begin{aligned} u_{\ell _1 \ell _2 i}= & {} \sum _{j=1}^4 \sum _{k_1=1}^2 x_{ijk_1}u_{\ell _1 j} u_{\ell _2 k_1} \end{aligned}$$

23 $$\begin{aligned} u_{\ell _4 \ell _5 i}= & {} \sum _{j=1}^4 \sum _{k_1=1}^2 x'_{ijk_1}u_{\ell _4 j} u_{\ell _5 k_1}. \end{aligned}$$

After identifying $u_{\ell _1 j}, u_{\ell _2 k_1}, u_{\ell _4 j}, \; \mbox{and} \; u_{\ell _5 k_1}$ is considered, *P*-values are attributed to *i*, as

24 $$\begin{aligned} P_i= & {} P_{\chi ^2} \left[ > \left( \frac{ u_{\ell _1 \ell _2 i}}{\sigma _{\ell _1 \ell _2}}\right) ^2 \right] , \end{aligned}$$

25 $$\begin{aligned} P_i= & {} P_{\chi ^2} \left[ > \left( \frac{ u_{\ell _4 \ell _5 i}}{\sigma _{\ell _4 \ell _5}}\right) ^2 \right] . \end{aligned}$$

The computed *P*-values were corrected by the BH criterion, and *i*s associated with adjusted P-values less than 0.01 were selected.

### Retrieval of gene symbols included in selected genomic regions

After selecting genomic regions, we needed to retrieve the gene symbols included in the selected genomic regions. This could be done using the biomaRt package implemented in R by specifying the hg19 human genome to which short reads were mapped.

### Ensembl gene ID to gene symbol

Since gene expression profiles are defined using Ensembl gene IDs, we needed to convert these IDs to gene symbols. This was done by uploading gene symbols selected by TD-based unsupervised FE to DAVID^27^. Uploaded Ensembl gene IDs were converted to gene symbols using the gene ID conversion tool implemented in DAVID by specifying the official gene symbol as the target of conversion.

### Enrichment analysis

Identified gene symbols were uploaded to Enrichr^28^, which is an enrichment server, to evaluate various enrichments within sets of identified gene symbols.

### Various conventional feature selections

#### Linear regression-based feature selection

To select genes or genomic regions using linear regression analysis, the ls function in the base package in R was used. *P*-values computed by ls were corrected by the BH criterion, and genes or genomic regions associated with adjusted *P*-values less than 0.01 or 0.05 were selected.

When linear regression was applied to gene expression, $x_{it}$,

26 $$\begin{aligned} x_{it} = a_i + b_i T(t) \end{aligned}$$

was assumed, where $T(1)=0, T(2)=6, T(3)=12, \; \mbox{and} \; T(4)=24$.

When linear regression was applied to m6A, $x_{ktj}$,

27 $$\begin{aligned} x_{ktj} = a_k + b_k T(t)j \end{aligned}$$

was assumed.

When linear regression was applied to the 1st synthetic data,

28 $$\begin{aligned} x_{ijk} = a_i + b_i jk \end{aligned}$$

was assumed.

#### SAM

When SAM^29^ was applied to gene expression, $x_{it}$, or m6A, $x_{ktj}$, are assumed to be classified into four classes based on *t* (for gene expression) or eight classes based on the combination of *t* and *j* (for m6A), respectively. The sam function was implemented in the siggenes package in R.

#### Limma

When limma^30^ was applied to gene expression, $x_{it}$, or m6A, $x_{ktj}$, respectively, they were assumed to be classified in the same way as in SAM. Limma was applied to logarithmically converted $x_{it}$ or $x_{ktj}$. The limma function was implemented in the limma package in R.

When limma was applied to the first synthetic data set, $x_{ijk}$ was classified into eight classes based on the pairs of *j* and *k*. Because $x_{ijk}$ takes both positive and negative values, $x_{ijk}$s themselves were regarded as logarithmically converted valRes.

#### Random forest

When random forest^31^ was applied to gene expression, $x_{it}$, or m6A, $x_{ktj}$, respectively, they are assumed to be classified in the same way as in SAM. When it was applied to the first synthetic data set, $x_{ijk}$ was classified into eight classes, as in the case of limma. The randomForest function was implemented in the randomForest package. Features included in OOB were selected by selecting features with non-zero importance given by the importance function implemented in the randomForest package in R.

## Supplementary Information

A sample R-code to perform analyses in this study.
